# Tofu processing wastewater as a low-cost substrate for high activity nattokinase production using *Bacillus subtilis*

**DOI:** 10.1186/s12896-021-00719-1

**Published:** 2021-10-07

**Authors:** Tao Li, Chenyi Zhan, Gege Guo, Zhaoxing Liu, Ning Hao, Pingkai Ouyang

**Affiliations:** College of Biotechnology and Pharmaceutical Engineering, State Key Laboratory of Materials-Oriented Chemical Engineering, Jiangsu National Synergetic Innovation Center for Advanced Materials (SICAM), Nanjing Tech University, Nanjing, 211816 China

**Keywords:** Tofu processing wastewater, *Bacillus subtilis*, Nattokinase, Media optimization

## Abstract

**Background:**

Even though tofu is a traditional Chinese food loved by Asian people the wastewater generated during the production of tofu can pollute the environment, and the treatment of this generated wastewater can increase the operating cost of the plant. In this study, the production of nattokinase could be achieved by using the nitrogen source in tofu processing wastewater (TPW) instead of using the traditional nattokinase medium. This meets the need for the low-cost fermentation of nattokinase and at the same time addresses the environmental pollution concerns caused by the wastewater. *Bacillus subtilis* 13,932 is, a high yielding strain of nattokinase, which is stored in our laboratory. To increase the activity of nattokinase in the tofu process wastewater fermentation medium, the medium components and culture parameters were optimized. Nattokinase with high enzymatic activity was obtained in 7 L and 100 L bioreactors when TPW was used as the sole nitrogen source catalyzed by *Bacillus subtilis*. Such a result demonstrates that the production of nattokinase from TPW fermentation using *B. subtilis* can be implemented at an industrial level.

**Results:**

The peptide component in TPW is a crucial factor in the production of nattokinase. Box–Behnken design (BBD) experiments were designed to optimize various critical components, i.e., Glucose, TPW, MgSO_4_·7H_2_O, CaCl_2_, in nattokinase fermentation media. A maximum nattokinase activity was recorded at 37 °C, pH 7.0, 70 mL liquid medium, and 200 rpm. The highest nattokinase activities obtained from 7 to 100 L bioreactors were 8628.35 ± 113.87 IU/mL and 10,661.97 ± 72.47 IU/mL, respectively.

**Conclusions:**

By replacing the nitrogen source in the original medium with TPW, there was an increase in the enzyme activity by 19.25% after optimizing the medium and culture parameters. According to the scale-up experiment from conical flasks to 100 L bioreactors, there was an increase in the activity of nattokinase by 47.89%.

**Supplementary Information:**

The online version contains supplementary material available at 10.1186/s12896-021-00719-1.

## Background

Being a serine proteinase, nattokinase has been widely used in the prevention and treatment of thrombosis-related disease due to its strong fibrin hydrolysis ability [1, 2]. Compared with urokinase and streptokinase, nattokinase is safer, more economical and it can be more easily absorbed by the body [3]. According to a recent survey, nearly 17 million people die each year from cardiovascular-related diseases [4]. As such, to address this critical issue, nattokinase has been extensively researched for its potential in treating these diseases. Nattokinase has presented a significant impact in the Chinese culture as a functional food or as an *intravenous* drug. Thus, to meet the market demand for nattokinase, it is highly vital to reduce the cost of nattokinase fermentation media and to increase the yield of nattokinase during production.

Currently, various low-cost raw material have been explored for the fermentation production of nattokinase [5, 6]. For instance, Ansuman Sahoo et al*.* [7] had reported the use of cheese whey as an alternative raw material for the production of nattokinase. It was shown that the nattokinase activity increased to 833.43 U/mL after being supplied with 10 g/L yeast extract, while achieving a corresponding reduction in the cost of the fermentation medium by 55–56%. Pan et al*.* [8] used cassava starch and soybean as their carbon and nitrogen sources for the production of the fibrinolytic enzyme. The fermentation process in a 100 L pilot fermenter was optimized, and a maximum fibrinolytic enzyme activity of 3787 U/mL was achieved. The fermentation of soybean residue as a component of the medium for the co-production of nattokinase and MK-7 was reported by Wang et al. [9]. It was shown that after the optimization of the response surface, the concentrations of MK-7 and nattokinase were able to reach 91.25 mg/L and 2675.73 U/mL, respectively. Thus, it can be concluded that the use of low-cost raw materials as substrates is a common and effective strategy in achieving an economical nattokinase fermentation.

Tofu processing wastewater (TPW) is a major by-product generated during the tofu preparation process, whereby approximately 7–10 kg of wastewater is produced per 1 kg of soybeans processed [10, 11]. Due to the high chemical oxygen demand (COD) and biochemical oxygen demand (BOD) content in TPW, it cannot be discharged directly into the environment [12, 13]. The nutrient-rich wastewater generated from tofu processing contains about 0.59 g/L total nitrogen, 0.078 g/L ammonia nitrogen, 0.26 g/L nitrate, and various metal ions, which provide suitable environments for the microorganisms to grow [14]. Hence, to reduce the risk of causing damage to the environment and humans, it is vital for the soy processing industry to recycle and treat TPW, even though this may result in increased production costs. In this study, nitrogen source in the original medium was replaced with wastewater generated from tofu processing. Based on this concept, Box–Behnken design (BBD) experiments were designed to optimize the content of each component in the medium. In addition to the optimization of components in the medium, environmental parameters were also optimized appropriately. To demonstrate the scalability of the as-proposed strategy in industrial-level wastewater utilization, the output of the nattokinase fermentation was scaled up from shake flask to 100 L bioreactor. The trend of the enzyme production from *Bacillus subtilis* fermentation under different conditions was also investigated.

## Results

### Comparison of different nitrogen sources on nattokinase activity and biomass

**Fig. 1:**
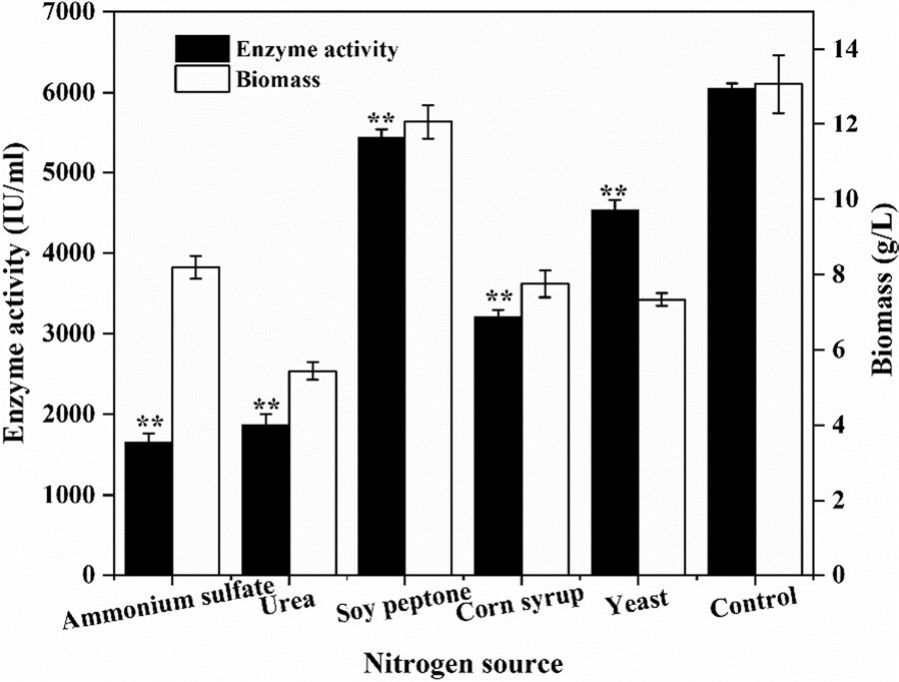
Comparison of the effects of different nitrogen sources on the synthesis of nattokinase. Control: 100% TPW as nitrogen source for blank control. *Significance code: *P* < 0.05; **Significance code: *P* < 0.01.

Soy peptone was added to the basal fermentation medium at a concentration of 25 g/L (about 9% of total nitrogen), while other organic and inorganic nitrogen were added with the same total nitrogen content of 2.25 g/L. The effects of TPW on the fermentative activity of nattokinase were compared with those of five other nitrogen sources, and the results are shown in Fig. 1. The highest nattokinase activity of 6045.58 ± 69.62 IU/mL was produced by TPW, which is far superior as compared to the results obtained from other sources. This is then followed by the enzyme activities produced by soy peptone, yeast powder, and corn pulp, whereby increased enzyme activities of 5429.25 ± 101.66 IU/mL, 4525.32 ± 133.207 IU/mL, and 3207.16 ± 87.05 IU/mL, respectively, are achieved.

In contrast, two inorganic nitrogen sources, i.e., (NH_4_)_2_SO_4_ and urea, are ineffective in the synthesis of nattokinase, as the nattokinase activities are less than 2000 IU/mL (Fig. 1).

### Effects of different amino acids and peptides on the fermentation of nattokinase

**Fig. 2:**
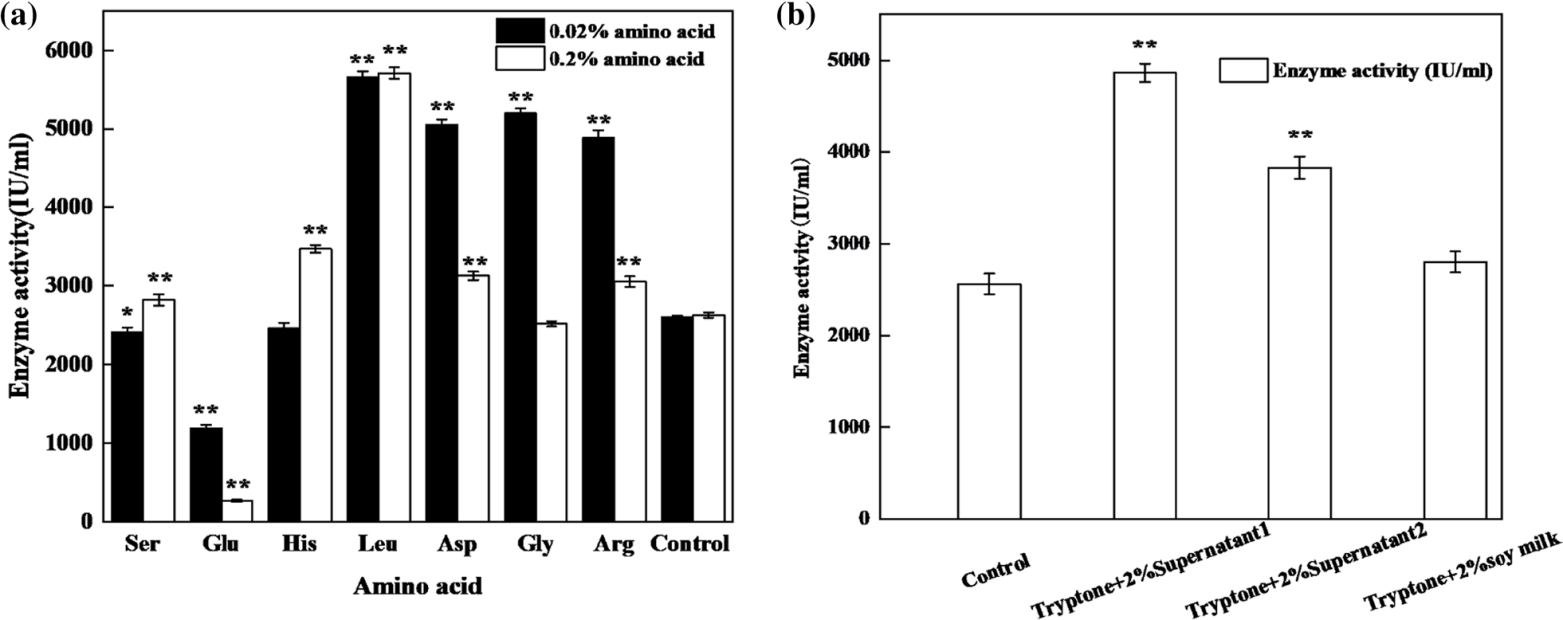
Effects of various amino acids (**a**) and soy milk hydrolysate (**b**) on the nattokinase activity. Control: 20 g/L Tryptone as nitrogen source for blank control. *Significance code: *P* < 0.05; **Significance code: *P* < 0.01.

The main nitrogen source components in TPW that are used for the growth of *Bacillus subtilis* include proteins, amino acids, peptides, etc. These components may induce an increase in the expression of nattokinase. As such, the primary amino acid components in TPW were determined using high-performance liquid chromatography (HPLC), and the results are shown in Table 1. It is revealed that TPW mainly contains eight amino acids, i.e., Asp, Glu, Arg, Ser, Gly, Leu, Lys, and His, with Asp, Glu, and Arg (> 1000 mg/L) having the highest concentrations; Ser and Gly (> 100 mg/L, < 1000 mg/L) having the second-highest concentrations; and the remaining amino acid fractions having lower concentrations (< 100 mg/L). Subsequently, to observe the effects of amino acid compositions on the fermentation of nattokinase, different types of amino acids were added to the control medium. As shown in Fig. 2a, the addition of Leu, Asp, Gly, and Arg at low concentrations of 0.2% can significantly increase the nattokinase activity. However, such an effect diminishes as the concentration of amino acid increases. Only Leu exhibit a more pronounced enhancement in the enzyme activity. In contrast, increasing the concentration of glutamate can increase the inhibition of nattokinase synthesis, which indicates that glutamate can exert a significant inhibitory effect on the fermentation of nattokinase. Figure 2b shows the significant effects of supernatant 1 and supernatant 2 on increasing the nattokinase activity. When compared with the control group, the activity of nattokinase after adding supernatant 1 and 2 increase by 89.99% and 49.53%, respectively. However, adding soymilk to the system shows a negligible effect on the increase in enzyme activity.

**Table 1:** Amino acid contents in each component

	Asp/mg/L	Ser/mg/L	Gly/mg/L	Arg/mg/L	Lys/mg/L	Glu/mg/L	His/mg/L	Leu/mg/L
Supernatant 1	36.12 ± 1.05	62.75 ± 2.83	22.01 ± 2.16	21.8 ± 1.21	15.55 ± 0.45	1072.88 ± 25.38	2670.45 ± 9.07	5.39 ± 0.79
Supernatant 2	15.34 ± 0.59	21.13 ± 1.80	8.63 ± 0.60	9.25 ± 0.88	4.71 ± 0.55	1061.16 ± 3.67	4044.52 ± 29.28	71.15 ± 1.55
TPW	2001.60 ± 5.09	955.23 ± 5.51	601.74 ± 3.28	601.90 ± 3.46	83.09 ± 2.66	1875.47 ± 19.78	124.59 ± 8.29	10.34 ± 1.06

### Optimization of nattokinase production using BBD

Based on the relevant literature and previous experimental results, various compounds such as glucose, TPW, MgSO_4_·7H_2_O, and CaCl_2_ had been confirmed to exert significant effects on enhancing the nattokinase activity. As such, to enhance the nattokinase activity, the fermentation medium was optimized by designing a 3-level BBD experiment based on 4-factor. Twenty-nine sets of experiments (with each repeated three times) were designed using BBD, and the results were validated using the actual and predicted values as summarized in Table 2. The results shown in Table 2 were analyzed with linear regression using Design Expert11 software, and the quadratic polynomial regression equation obtained by standard ANOVA was;

$$\begin{aligned} & 7045.27{-}433.70{\text{A}}\, + \,2460.93{\text{B}} - 47.23{\text{C}}\, + \,137.04{\text{D}} \\ & \quad - 46.92{\text{AB}}\, + \,155.16{\text{AC}}\, + \,473.72{\text{AD}} - 63.68{\text{BC}} \\ & \quad - 51.17{\text{BD}}\, + \,229.87{\text{CD}} - 1557.49{\text{A}}^{2} - 1597.73{\text{B}}^{2} \\ & \quad - 256.20{\text{C}}^{2} - 444.94{\text{D}}^{2} . \\ \end{aligned}$$

**Table 2:** Experimental design and results of the Box-Behnken Design

	Factor 1	Factor 2	Factor 3	Factor 4	Enzyme activity (IU/mL)	
Run	A: Glucose (g/L)	B: TPW (%)	C: MgSO_4_·7H_2_O (g/L)	D: CaCl_2_ (g/L)	Actual value	Predicted value
1	22.5	100	1	1	7472.70 ± 137.28	7549.40
2	22.5	75	0.5	0.1	6863.40 ± 84.29	6484.19
3	22.5	100	0.5	0.55	7719.21 ± 69.39	7763.18
4	35	75	1	1	4637.23 ± 32.82	5219.90
5	10	75	0.5	0.55	5608.72 ± 118.44	5867.68
6	22.5	75	1	0.55	7202.28 ± 72.56	7045.27
7	22.5	50	1	1	2552.45 ± 40.53	2729.88
8	22.5	75	0.5	1	6631.05 ± 31.61	6298.53
9	22.5	100	1.5	0.55	7563.01 ± 50.01	7541.37
10	22.5	75	1	0.55	7306.88 ± 48.21	7045.27
11	22.5	50	1.5	0.55	2685.39 ± 156.66	2746.88
12	35	75	1.5	0.55	4927.65 ± 94.96	4905.82
13	22.5	75	1.5	1	6627.17 ± 75.44	6663.81
14	22.5	75	1	0.55	7163.78 ± 46.64	7045.27
15	35	100	1	0.55	6522.90 ± 55.96	5870.37
16	22.5	75	1	0.55	6900.12 ± 49.35	7045.27
17	10	75	1	1	5680.77 ± 71.76	5139.86
18	10	75	1.5	0.55	5507.49 ± 66.73	5462.89
19	10	50	1	0.55	1505.96 ± 69.34	1815.91
20	22.5	50	0.5	0.55	2586.87 ± 60.69	2713.96
21	10	100	1	0.55	6337.79 ± 52.37	6831.60
22	22.5	75	1	0.55	6653.31 ± 42.06	7045.27
23	35	50	1	0.55	1878.74 ± 55.13	1042.35
24	35	75	0.5	0.55	4408.22 ± 78.25	4689.94
25	22.5	75	1.5	0.1	5940.07 ± 78.91	5930.00
26	10	75	1	0.1	6290.44 ± 87.9	5813.23
27	22.5	50	1	0.1	2193.04 ± 137.11	2353.47
28	22.5	100	1	0.1	7317.97 ± 126.32	7377.67
29	35	75	1	0.1	3352.02 ± 175.91	3998.39

**Fig. 3:**
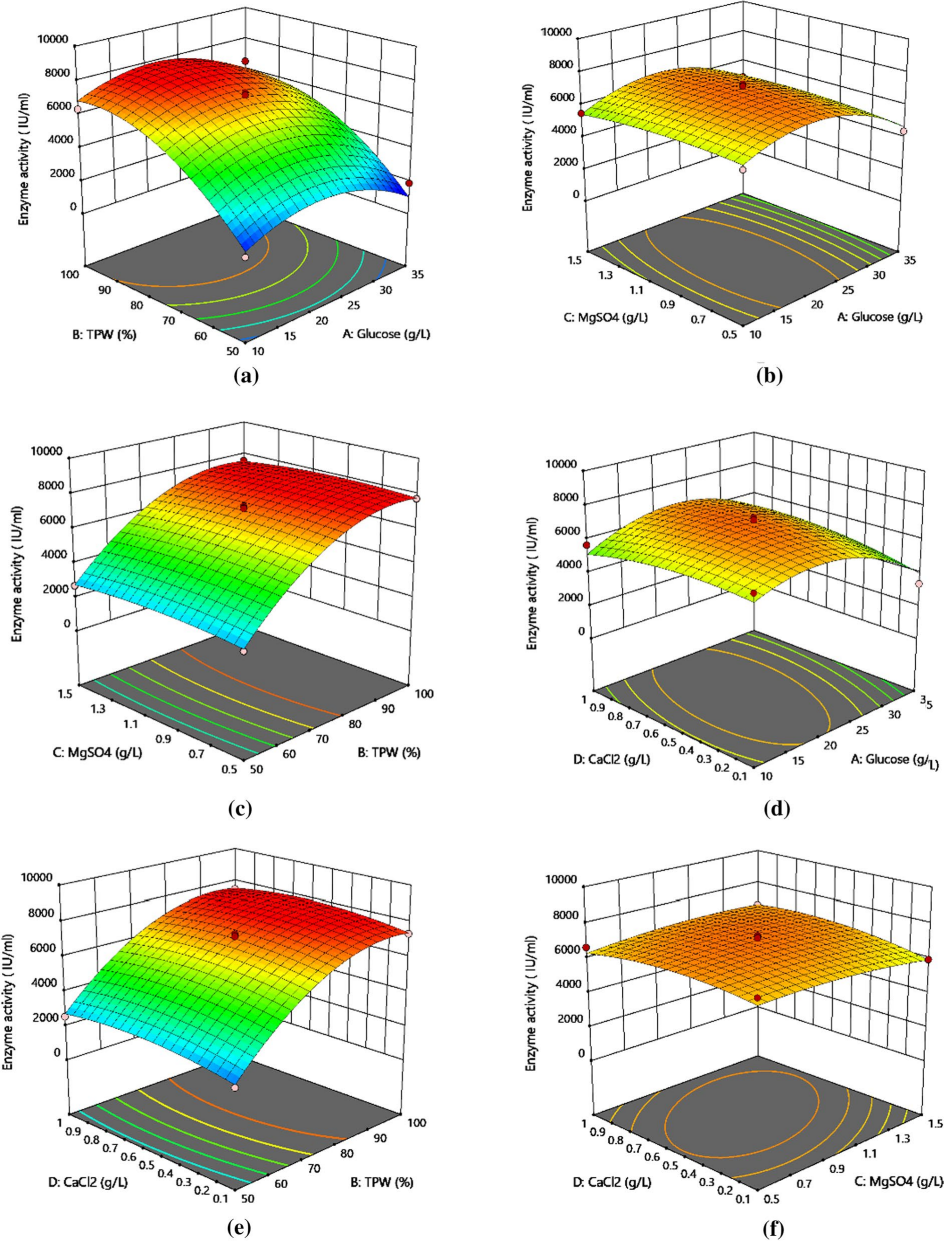
Response surface curves showing the effects of each component in the medium on the production of nattokinase. The response curves show the interaction between different factors. **a** Interaction between TPW and glucose; **b** Interaction between MgSO_4_ and glucose; **c** Interaction between MgSO_4_ and TPW; **d** Interaction between CaCl_2_ and glucose; **e** Interaction between CaCl_2_ and TPW; **f** Interaction between MgSO_4_ and CaCl_2_

According to the ANOVA results presented in Table 3, the model used is statistically significant as the model significance test *P* value is less than 0.05, while the *P* value of the misfit term is 0.0775 (which is insignificant since it is more than 0.05). Figure 3 shows the relationships between the variables using 3D response surface plots. Based on Table 3, nattokinase fermentation is significantly affected by glucose (0.0096 < 0.05) and TPW (< 0.0001). However, according to the 3D response surface plots (Fig. 3), no obvious relationship between the variables can be observed. It is worth noting that nattokinase activity decreases with decreasing TPW concentration, which indicates the vital role of TPW in achieving nattokinase with high enzymatic activity. It can be observed that the 3D response surface plots for all three variables are convex, except for TPW. At this point, there is an increase in the nattokinase activity with increasing concentrations of TPW, MgSO_4_·7H_2_O, and CaCl_2_ until a maximum nattokinase activity is achieved. After which, there is a decrease in the enzyme activity with increasing concentrations of each variable. The experimental validation of nattokinase activity based on the optimal combination of fermentation media is slightly higher than that of the predicted value (7092.5 IU/mL), which suggests the reliability of the model. Thus, based on the results, the optimal medium combination consists of 30.868 g/L glucose, 93.669% TPW, 1.129 g/L MgSO_4_·7H_2_O, and 0.791 g/L CaCl_2_.

**Table 3:** ANOVA result based on a quadratic model for the activity of nattokinase produced by *Bacillus subtilis* 13932

Source	Sum of Squares	df	Mean Square	F-value	*P* value	
Model	1.040E + 08	14	7.429E + 06	29.56	< 0.0001	Significant
A-Glucose*	2.257E + 06	1	2.257E + 06	8.98	0.0096	
B-TPW*	7.267E + 07	1	7.267E + 07	289.17	< 0.0001	
C-MgSO_4_	26,763.25	1	26,763.25	0.1065	0.7490	
D-CaCl_2_	2.253E + 05	1	2.253E + 05	0.8967	0.3597	
AB	8804.90	1	8804.90	0.0350	0.8542	
AC	96,304.36	1	96,304.36	0.3832	0.5458	
AD	8.976E + 05	1	8.976E + 05	3.57	0.0797	
BC	16,221.98	1	16,221.98	0.0645	0.8031	
BD	10,473.36	1	10,473.36	0.0417	0.8412	
CD	2.114E + 05	1	2.114E + 05	0.8410	0.3746	
A^2^*	1.573E + 07	1	1.573E + 07	62.61	< 0.0001	
B^2^*	1.656E + 07	1	1.656E + 07	65.89	< 0.0001	
C^2^	4.258E + 05	1	4.258E + 05	1.69	0.2141	
D^2^	1.284E + 06	1	1.284E + 06	5.11	0.0403	
Residual	3.518E + 06	14	2.513E + 05			
Lack of Fit	3.237E + 06	10	3.237E + 05	4.59	0.0775	Not significant
Pure Error	2.818E + 05	4	70,460.26			
Cor Total	1.075E + 08	28				

^*^ Significant at *P* < 0.01

### Optimization of fermentation conditions for the production of nattokinase

**Fig. 4:**
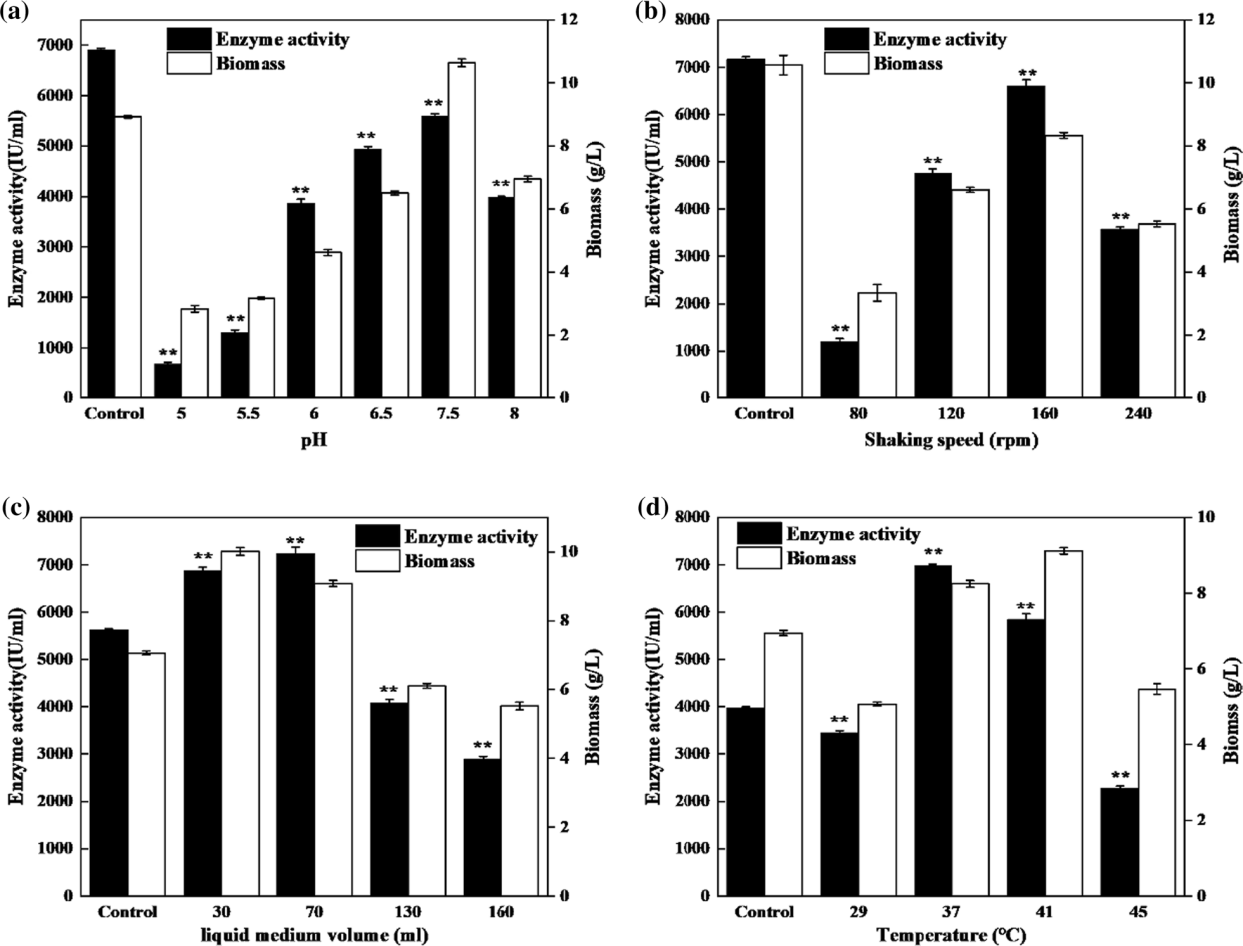
Effects of initial pH (**a**), shaking speed (**b**), liquid medium volume (**c**), and temperature (**d**) on nattokinase activity. **a** Control: pH 7, **b** Control: shaking speed 200 rpm, **c** Control: liquid medium volume 100 mL/500 mL, **d** Control: temperature 33 °C. *Significance code: *P* < 0.05; **Significance code: *P* < 0.01

Figure 4a illustrates the effects of initial pH, i.e., from 5 to 8, on the nattokinase activity of strain *Bacillus subtilis* 13932. According to the result, the highest enzyme activity (6900.04 ± 40.05 IU/mL) and the highest biomass (10.63 ± 0.12 g/L) were obtained at the pH range of 7–7.5. Meanwhile, varying degrees of reduction in the enzyme activity and biomass were observed at other pH values. Figure 4b shows the amount of nattokinase produced by the strain *Bacillus subtilis* 13932 at pH 7 with different shaking speeds, i.e., 80, 120, 160, 200, and 240 rpm. Based on the result, the nattokinase activity and the bacterial biomass were significantly enhanced with increasing shaking speeds, whereby the highest nattokinase activity of 7167.42 ± 49.59 IU/mL was achieved at 200 rpm. However, with increasing shaking speed beyond 200 rpm, the nattokinase activity was reduced to 3579.01 ± 44.17 IU/mL. To evaluate the nattokinase activity at various medium volumes, the bacterial biomass of *Bacillus subtilis* and nattokinase activity were measured at medium volumes of 30/500 mL, 70/500 mL, 100/500 mL, 130/500 mL, and 160/500 mL. As shown in Fig. 4c, an optimal nattokinase activity of 7233.35 ± 140.90 IU/mL was obtained at a medium volume of 70 mL. Interestingly, as the medium volume increased from 70 to 160 mL, there was a constant reduction in the nattokinase activity. Figure 4d shows the gradual increase in the bacterial biomass and enzyme activity with increasing temperatures (when the fermentation temperature was in the range of 29–37 °C). As the temperature increased beyond 37 °C, both the enzyme production and dry weight of bacteria exhibited further reduction. Thus, based on the collective results, it can be concluded that 37 °C is the optimal temperature for the growth of *Bacillus subtilis* (8.24 ± 0.09 g/L) and enzyme production (6984.51 ± 26.25 IU/mL).

### Cost and scaling up of batch fermentation

**Fig. 5:**
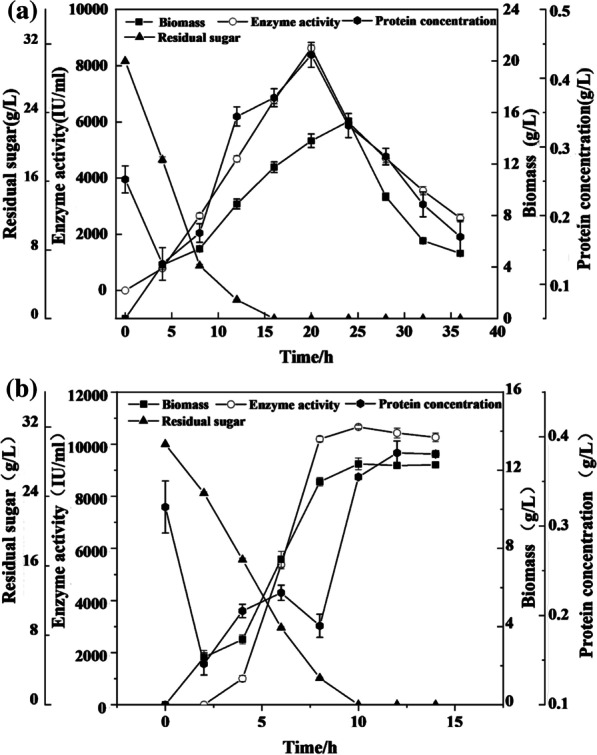
Batch fermentation in 7 L and 100 L bioreactors

It has been shown through the optimization experiments (based on shake flask set-up) that *Bacillus subtilis* 13,932 can ferment TPW to produce nattokinase with high enzymatic activity. To demonstrate the scalability and reliability of the fermentation process, scale-up experiments involving 7 L and 100 L bioreactors were conducted, and the results are shown in Fig. 5a and b, respectively. The highest nattokinase activity of 8628.365 ± 113.87 IU/mL is achieved in the 7 L bioreactor after 20 h. In contrast, the maximum nattokinase activity in the 100 L bioreactor is achieved after only 10 h, and there is a 23.56% increase in the nattokinase activity as compared to that recorded in the 7 L bioreactor. There is a rapid consumption of glucose during the fermentation process, with almost fully depleted residual sugar contents in both bioreactors after 10–12 h. It can be observed that the biomass gradually reaches a maximum as the glucose is depleted. In the 7 L bioreactor, the biomass after 24 h reaches a maximum of 15.32 ± 0.24 g/L. As the fermentation system is expanded to 100 L, the maximum biomass of the bacteria reaches a maximum of 12.33 ± 0.29 g/L after 10 h. Table 4 compares the cost of the nattokinase fermentation media and their corresponding maximum nattokinase activities from various literature. According to Table 4, higher nattokinase activity is obtained by replacing the nitrogen source in the original medium with TPW, when compared to other comparable literature. Furthermore, the cost of the as-proposed fermentation medium based on TPW is as low as US$19, whereas the costs of other nattokinase fermentation media reported in the literature were at least US$20.

**Table 4:** Cost of nitrogen source and medium required for the production of nattokinase

Nitrogen source	Culture mode	Maximum nattokinase activity	References	Nitrogen source cost (Ton/dollar)	Medium cost (Ton/dollar)
10 g/L yeast extract	200 mL flask	12.34 FU/mL	Wang et al. [29]	30–35	31.97–39.02
13 g/L soy peptone	250 mL flask	31.06 ± 0.297 FU/mL	Nguyen [30]	52.22–63.41	64.6–81.25
20 g/L soy peptone	100 mL flask	871.56 IU/mL	Zhou et al. [31]	80.33–97.55	90.71–117.13
10 g/L peptone + 5 g/L yeast extract	250 mL flask	3284 ± 58 IU/mL	Ju et al. [32]	55.16–66.27	78.10–89.21
55 g/L peptone	250 mL flask	3194.25 U/mL	Deepak et al. [33]	275.31–314.64	281.8–326.17
100%TPW	500-mL flask	7708.89 ± 133.67 IU/mL	In study	–	19–37.92

## Discussion

Nattokinase is produced during the fermentation of *Bacillus subtilis*, and it possesses good fibrinolysis property [15]. In this study, TPW was used as an alternative raw fermentation medium for *Bacillus subtilis* to produce nattokinase. The highest viability of nattokinase can be achieved by replacing the nitrogen source in the original medium with TPW. It is shown that organic nitrogen is significantly more effective than inorganic nitrogen in promoting the synthesis of nattokinase. To satisfy the cell growth requirements, complex nitrogen sources have to be broken down by increasing the expression of proteases such as nattokinase by *Bacillus subtilis* [16]. In addition to the high concentration of COD and BOD (Table 5), the main components of TPW include proteins, peptides, and amino acids too. The primary amino acid components of TPW, i.e., Asp, Ser, Gly, Arg, Lys, Glu, His, and Leu, were added to a blank medium separately, and the results show that the addition of certain low amino acids concentrations (Leu, Arg, Asp, Gly) are effective in enhancing the activity of nattokinase.

**Table 5:** Chemical parameters of TPW

Parameter	Value
pH	5.02 ± 0.22
Total sugar (g/L)	4.9 ± 1.05
Total nitrogen (g/L)	0.91 ± 0.36
NH_4_^+^-N (g/L)	0.026 ± 0.005
Total phosphorus (g/L)	0.127 ± 0.017
BOD (g/L)	23.7 ± 2.17
COD (g/L)	46 ± 3
Solid content (g/L)	0.045 ± 0.012
Cu^2+^ (mg/L)	0.73 ± 0.18
Zn^2+^ (mg/L)	0.29 ± 0.13

In contrast, as the concentration of amino acids added in the system increases, the other three amino acid concentrations (Arg, Asp, Gly), except Leu, exhibt detrimental effects on the synthesis of nattokinase. Thus, it can be hypothesized that the synthesis of nattokinase and enzyme activity can be inhibited by the presence of high amino acid concentration. Due to the high amino acid concentrations in TPW, the role of amino acid-induced enzyme production in TPW can be excluded. This standpoint is consistent with other researchers, such as Li–Li Man, who suggested that the peptide component in the soybean peptone was a critical factor in facilitating the synthesis of nattokinase rather than amino acids [17].

To further investigate the role of peptides, soymilk was treated with acid hydrolysis during ethanol precipitation. This process can remove the soymilk proteins after the hydrolysis, which leaves behind the peptide fraction. 2% soymilk hydrolysate and 2% soymilk protein were added to the blank medium, and the results are shown in Fig. 2b. It can be observed that the addition of 2% soymilk hydrolysate is effective in promoting the production of nattokinase. Such a result indicates that the hydrolyzed peptide fraction is able to induce the production of nattokinase. TPW is the wastewater produced when soymilk is brined and extruded to form tofu. As the result of this physical action, free polypeptides can be generated, and this may be the key reason for the enhanced production of nattokinase from TPW.

According to the previous reports, glucose, Ca^2+^, and Mg^2+^ have been identified as key factors that can affect the production of nattokinase by *Bacillus subtilis* [18, 19]. Thus, it is necessary to optimize the concentrations of the key components in the TPW medium. BBD experiments were conducted to optimize the concentrations of the four key components, *i.e.*, glucose, TPW, Ca^2+^, and Mg^2+^, and the results show that the optimal combination consists of 30.868 g/L glucose, 93.669% TPW, 1.129 g/L MgSO_4_·7H_2_O, and 0.791 g/L CaCl_2_. Based on this optimal combination, the nattokinase activity is able to attain 7209.15 ± 195.46 IU/mL. According to the ANOVA results, glucose and TPW exert significant effects on the production of nattokinase. Other than the optimization of the components in the medium, culture conditions such as temperature, initial pH, loading volume, and speed can also influence the production of enzyme, and these conditions have to be optimized as well. Based on the results, an initial pH range of 7–7.5 is the most favorable for the production of nattokinase. The transportation of substances across the cell membrane of the microbe and various enzymatic reactions can be affected as the initial pH varies [20]. At the approporiate pH range, the relative metabolic efficiency of microorganisms can be enhanced. The growth of *Bacillus subtilis* is partially coupled with the production of nattokinase, and therefore achieving an efficient metabolic efficiency can promote both the growth of organism and enzyme production capacity. It is worth noting that shaker speed and liquid medium volume correlate with the dissolved oxygen (DO) concentration in the fermentation broth. Based on the result, the highest nattokinase activity is obtained with a shaker speed of 200 rpm or a liquid medium volume of 70 mL. An moderate oxygen level is necessary as high or low dissolved oxygen levels are not conducive to cell growth and enzyme production. This is because insufficient dissolved oxygen content in the fermentation medium can retard the growth of *Bacillus subtilis*, and this is detrimental towards the production of enzyme. On the other hand, too much dissolved oxygen can lead to oxygen toxicity in the bacterium, which also negatively affects enzyme production [21]. The most suitable enzyme production temperature for *Bacillus subtilis* is 37 °C. It can be observed that the enzyme activity decreases when the temperature deviates from 37 °C. As such, based on this result, it can be concluded that temperature can correlate with the catalytic activity of various enzymes in the microorganisms. The inhibition or inactivation of various enzymatic reactions in the microorganisms at unsuitable temperatures can affect their growth and the synthesis of nattokinase by secretion [22]. According to the result obtained from the scale-up fermentation experiments from 7 to 100 L bioreactors, there is a reduction in the fermentation cycle from 20 to 10 h, with a 23.5% increase in the nattokinase activity. When compared to the experiment based on shake flask, there is an increase in the enzyme production capacity of *Bacillus subtilis* in both scale-up fermentation experiments. Such a result may be due to the different forms of agitation used in the process and increased dissolved oxygen content in the fermentation broth. Thus, according to the collective results, the concept of using TPW at an industrial scale to replace the nitrogen source in the original culture medium for the production of nattokinase is highly feasible.

## Conclusions

Cardiovascular-related disease possesses significant threats to human health. Nattokinase has been proposed in the prevention and treatment of cardiovascular-related diseases such as hypertension due to its excellent fibrinolytic activity. To achieve a broader commercial application, it is highly necessary to produce nattokinase with high enzyme activity a low-cost. This study has confirmed the feasibility of producing nattokinase from the use of active fraction in tofu processing wastewater under the action of *Bacillus subtilis*. According to the results, only glucose, calcium, and magnesium ions were required in TPW for the production of nattokinase via fermentation. It was shown that *Bacillus subtilis* could utilize TPW medium better than soy peptone medium to produce the enzyme. After analyzing the factors that could induce the production of enzyme in TPW, it was noted that a certain peptide fraction from the hydrolysis of soy protein could contribute to the enhancement of nattokinase activity. Based on this foundation, the conditions of the enzyme production using *Bacillus subtilis* were optimized, and improved fermentation enzyme production activity was recorded. Furthermore, the feasibility of large-scale conversion of TPW for the production of nattokinase was demonstrated by conducting pilot tests in 7 L and 100 L bioreactors.

## Materials and methods

### Strain and material

Nattokinase-producing bacterium, i.e., *Bacillus subtilis* 13932 (Conservation No. CGMCC13932), was isolated from Chinese tempeh, and then it was subsequently conserved in the China General Microbiological Culture Collection Center. Fibrinogen (130 mg/bottle), thrombin (190 bp/bottle), and urokinase (1280 IU/bottle) standards were purchased from Beijing Zhongke Quality Control Biotechnology Co., Ltd. Experimental tofu processing wastewater and soy milk was provided by Nanjing Dou Guo Guo Food Technology Co. Other analytical reagents were purchased from Nanjing WANQING chemical Glassware and Instrument Co., Ltd.

### Basic culture media

100 mL LB liquid medium in a 500 mL Erlenmeyer flask was used as the seed solution, and it was incubated for 16 h before transferring 2% bacterial seed liquid to the tofu processing wastewater fermentation medium. The composition of blank medium was 20 g/L glucose, 20 g/L soybean peptone, 0.2 g/L CaCl_2_, and 0.6 g/L MgSO_4_·7H_2_O, with a pH of 7.0. Tofu processing wastewater replaces soy peptone as a nitrogen source, and henceforth referred as TPW culture medium.

### Pre-treatment of soy milk

The pH of the soy milk was adjusted to 5.5 by adding hydrochloric acid. After which, the mixture was stirred for 50 min at 50 °C. Subsequently, the mixture was centrifuged at 4000 rpm for 30 min to collect the supernatant (denoted as supernatant 1) for further experiment. 70% ethanol was then added to supernatant 1 and it was stirred for 30 min at 55 °C. Finally, the mixture was centrifuged to obtain supernatant 2 for further experiment [23].

### Tofu processing wastewater as an alternative nitrogen source for the production of nattokinase

A 3-level Box-Behnken Design (BBD) with 4-factor was conducted to optimize the effects of glucose, TPW, CaCl_2_, and MgSO4·7H_2_O on the production of nattokinase. The experimental design is shown in Table 2.

All experiments were repeated 3 times in conical flasks (500 mL) containing 100 mL liquid medium. Fermentation was performed at 33 °C, initial pH of 7.0–7.2, and 200 rpm for 60 h. At the end of the fermentation, nattokinase activity was recorded.

### Optimization of the fermentation conditions

Single-factor experiments were designed to optimize the culture conditions based on the optimized media. Fermentation was conducted at 33 °C and 100 mL/500 mL liquid medium volume for 60 h. The effects of the shaker speed (80, 120, 160, 200, and 240 rpm) and initial pH (5.5, 6.0, 6.5, 7.0, 7.5, and 8.0) on the production of nattokinase by *Bacillus subtilis* were assessed. The effects of temperature (29, 33, 37, 41, and 45 °C) and volume of liquid medium (30, 70, 100, 130, and 160 mL) on the viability of nattokinase were assessed at an initial pH of 7.0 and a shaker speed of 200 rpm.

### Batch fermentation

Pilot experiments involving a 7 L bioreactor were performed with a liquid medium volume of 3 L, a ventilation ratio of 1.1 vvm, and a speed of 300 rpm (To meet the demand for dissolved oxygen, the stirring speed in the 7 L bioreactor was increased to 300 rpm). Pilot experiments involving a 100 L bioreactor were performed with a liquid medium volume of 60 L, a ventilation ratio of 1.1 vvm, and a speed of 300 rpm (To meet the demand for dissolved oxygen, the stirring speed in the 100 L bioreactor was increased to 300 rpm). The incubation temperature and initial pH used in all cases were 37 °C and 7–7.5, respectively.

### Analysis methods

#### Biomass

The fermentation broth was centrifuged at 12,000 rpm for 15 min. After which, the collected residue was washed 3 times with saline (0.9% NaCl), and then it was dried in a vacuum drying oven at 55 °C until a constant weight was achieved.

#### TPW biochemical fraction analysis

The contents of COD, ammonia nitrogen, total phosphorus, Cu^2+^, and Zn^2+^ in the wastewater were analyzed using a multi-parameter water quality tester 5B-3BW (Beijing Lianhua Technology Development Co., Ltd.) [24, 25]. The contents of total nitrogen in the wastewater were analyzed using Kjeldahl (FOSS ScinoCo.,Ltd.). The pH of TPW was measured using a pH meter (METTLER TOLEDO), and the BOD was measured using a biochemical oxygen demand (BOD5) tester (Beijing Lianhua Technology Development Co.). Amino acids were determined by the pre-column derivatization with phenyl isothiocyanate [26].

### Enzyme activity determination method

Nattokinase activity was determined by employing the fibrin plate method [27, 28]. Urokinase was used as a standard. 39 mL of 1.5% (w/v) agarose dissolved in a phosphate-sodium chloride mixture (1:17 mixture consisting of phosphate buffer with pH of 7.8 and saline) was mixed with 39 mL of 1.5 mg/mL fibrinogen solution, and 3 mL of thrombin (1 bp/mL) solution was added and then it was mixed rapidly. 20 mL of the mixture was added to a 90 mm cell culture plate and it was left to solidify at room temperature for 1 h. The fermentation broth was centrifuged at 12,000 rpm for 5 min and then it was diluted 20-fold with saline (0.9% NaCl). The wells were punched on a fibrin plate, and 10 μL of enzyme solution was added to the wells. The system was incubated at 37 °C for 18 h. The vertical diameters of the fibrinolytic circles were determined by using vernier calipers. The enzymatic activity of the urokinase standard was used as the vertical coordinate, while the product of the two vertical diameters was used as the horizontal coordinate. The nattokinase activity was calculated based on the urokinase standard curve.

## Supplementary Information

**Additional file 1: Table S1.** Market prices of various carbon and nitrogen sources on Alibaba.com in March. **Figure S1.** High-performance liquid chromatography diagram of amino acid standard.

## Data Availability

All data generated or analyzed during this study are included in this published article and its Additional file 1.

## Abbreviations

TPW: Tofu process wastewater
BBD: Box–Behnken design
DO: Dissolved oxygen
vvm: Air volume/culture volume/min
HPLC: High-performance liquid chromatography
Asp: Aspartic acid
Ser: Serine
Gly: Glycine
Arg: Arginine
Lys: Lysine
Glu: Glutamic acid
His: Histidine
Leu: Leucine
COD: Chemical oxygen demand
BOD: Biochemical oxygen demand
PITC: Phenyl isothiocyanate
CGMCC: China general microorganism culture collection
