# Supplementary material for: Tofu processing wastewater as a low-cost substrate for high activity nattokinase production using Bacillus subtilis
Source: BMC Biotechnol. 2021 Oct 7;21:57. doi: 10.1186/s12896-021-00719-1 (PMC8499530; doi:10.1186/s12896-021-00719-1)

| Component | Industrial raw material prices (kg/$) |
| --- | --- |
| Glycerol | 0.837 |
| Glucose | 0.21-0.47 |
| Peptone | 5-5.71 |
| Soy peptone | 4.04-4.91 |
| Yeast power | 2.30-2.88 |
| Yeast extract | 3-3.5 |
| Na_2_HPO_4_ | 1.37-2.59 |
| NaH_2_PO_4_ | 1.44-2.16 |
| K_2_HPO_4_ | 1.73-2.88 |
| KH_2_PO_4_ | 1.44-2.88 |
| MgSO_4_·7H_2_O | 0.71-1.57 |
| CaCl_2_ | 0.57-0.72 |
| NaCl | 1.29-1.44 |

**Table1. Industrial raw material prices**

(according to the market prices in March 2021 from Alibaba Group)

**Fig. 1 High-Performance Liquid Chromatography Diagram of Amino Acid Standard**


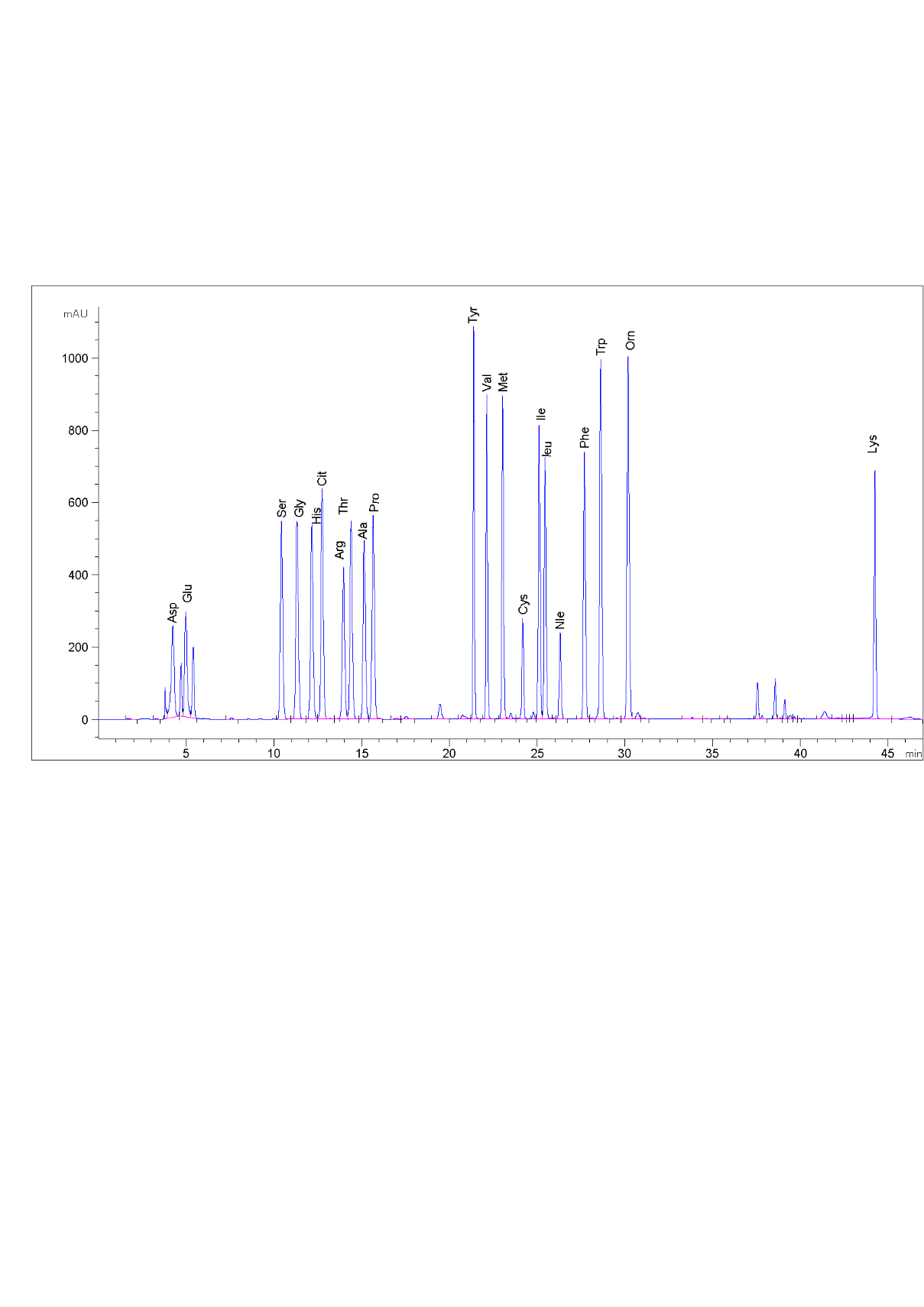

Supplement: Supplementary file 1 — Additional file 1: Table S1. Market prices of various carbon and nitrogen sources on Alibaba.com in March. Figure S1. High-performance liquid chromatography diagram of amino acid standard. [file 12896_2021_719_MOESM1_ESM.docx]
